# Machine learning based models for prediction of subtype diagnosis of primary aldosteronism using blood test

**DOI:** 10.1038/s41598-021-88712-8

**Published:** 2021-05-04

**Authors:** Hiroki Kaneko, Hironobu Umakoshi, Masatoshi Ogata, Norio Wada, Norifusa Iwahashi, Tazuru Fukumoto, Maki Yokomoto-Umakoshi, Yui Nakano, Yayoi Matsuda, Takashi Miyazawa, Ryuichi Sakamoto, Yoshihiro Ogawa

**Affiliations:** 1 Department of Medicine and Bioregulatory Science, Graduate School of Medical Sciences, Kyushu University, 3-1-1 Maidashi Higashi-ku, Fukuoka, 812-8582 Japan; 2 Department of Diabetes and Endocrinology, Sapporo City General Hospital, Sapporo, Japan

**Keywords:** Adrenal gland diseases, Adrenal glands

## Abstract

Primary aldosteronism (PA) is associated with an increased risk of cardiometabolic diseases, especially in unilateral subtype. Despite its high prevalence, the case detection rate of PA is limited, partly because of no clinical models available in general practice to identify patients highly suspicious of unilateral subtype of PA, who should be referred to specialized centers. The aim of this retrospective cross-sectional study was to develop a predictive model for subtype diagnosis of PA based on machine learning methods using clinical data available in general practice. Overall, 91 patients with unilateral and 138 patients with bilateral PA were randomly assigned to the training and test cohorts. Four supervised machine learning classifiers; logistic regression, support vector machines, random forests (RF), and gradient boosting decision trees, were used to develop predictive models from 21 clinical variables. The accuracy and the area under the receiver operating characteristic curve (AUC) for predicting of subtype diagnosis of PA in the test cohort were compared among the optimized classifiers. Of the four classifiers, the accuracy and AUC were highest in RF, with 95.7% and 0.990, respectively. Serum potassium, plasma aldosterone, and serum sodium levels were highlighted as important variables in this model. For feature-selected RF with the three variables, the accuracy and AUC were 89.1% and 0.950, respectively. With an independent external PA cohort, we confirmed a similar accuracy for feature-selected RF (accuracy: 85.1%). Machine learning models developed using blood test can help predict subtype diagnosis of PA in general practice.

## Introduction

Primary aldosteronism (PA) is a common cause of secondary hypertension, accounting for approximately 5% to 10% of all patients with hypertension^1,2^. Patients with PA have higher cardiovascular morbidity and mortality than age- and sex-matched patients with essential hypertension^3,4^. Furthermore, those with unilateral subtype of PA are at an increased risk of cardiometabolic disease relative to those with bilateral subtype of PA, thus requiring a more specific treatment adrenalectomy, rather than drug therapy^5–7^. According to the Endocrine Society clinical practice guideline^8^, the diagnosis of PA is hierarchical: case detection, case confirmation, and subtype classification. Case detection is usually performed in general practice, whereas case confirmation and subtype diagnosis are largely performed in specialized centers. However, it was reported that the rate of aldosterone and renin measurements in hypertensive patients in general practitioners was relatively low (7%-8%) and that patients with suspected PA were often referred to cardiologists rather than to endocrinologists or hypertension centers^9^. This may be partly due to the lack of available clinical models using routine blood test in the general practice, such as general practitioners, cardiologists, and nephrologists. Therefore, availability of simple and early prediction of unilateral subtype of PA in the general practice is desirable to facilitate sending patients to appropriate specialized facilities and increasing the diagnostic rate of unilateral subtype of PA.

Machine learning provides techniques that can automatically build computational models of complex relationships between observable and relevant objective variables by processing available data and maximizing performance criteria^10^. Approaches using machine learning for predicting new data from identified patterns has helped detect patterns that are difficult to recognize from complex combinations of multiple biomarkers^11^. However, the analytical approaches for PA have not been fully applied, and when combined with clinical data, could lead to the development of new models to predict unilateral subtype of PA.

The aim of this study was to develop optimal models based on machine learning analysis for predicting unilateral subtype of PA from clinical data available in general practice. The validity of our models was confirmed by an external PA cohort.

## Materials and methods

### Development of predictive models for subtype diagnosis of PA

#### Modeling PA cohort

In this study, we consecutively included 253 patients with PA who were referred to Kyushu University Hospital and who underwent adrenal venous sampling (AVS) with adrenocorticotropic hormone (ACTH) stimulation for the purpose of subtype diagnosis, between January 2007 and March 2020. This study was part of the Kyushu Adrenal Network Database for Advanced medicine (Q-AND-A) study^12^. All patients were diagnosed with PA according to the guidelines of the Japan Endocrine Society and Japanese Society of Hypertension^13,14^, with case detection and confirmatory tests. This retrospective study was approved by the institutional review board of Kyushu University (approval study number: 2019–526) and was performed in accordance with relevant guidelines. Informed consent was obtained from the patients upon admission to our hospital.

#### PA confirmation and subtype criteria

PA was diagnosed with at least one positive confirmatory test; saline infusion test or captopril challenge test based on the methods and criteria described in the above guidelines. More than two weeks prior to the diagnosis, antihypertensive drugs were routinely changed to calcium channel blockers and/or α-adrenergic blockers. Oral potassium was generally supplemented in patients with hypokalemia. In AVS with ACTH stimulation, adrenal vein cannulation was considered successful when the ratio of cortisol concentration in the adrenal vein to that in the inferior vena cava was > 5^15^. We considered unilateral lateralization, when the aldosterone-to-cortisol ratio on the dominant adrenal side was at least four times higher than that on the non-dominant side^15^. Apparent bilateral aldosterone suppression was defined when the aldosterone-to-cortisol ratios in both the dominant and non-dominant adrenal veins were lower than that in the inferior vena cava^16,17^.

#### Definition of clinical and biochemical outcomes

Biochemical and clinical outcomes after unilateral adrenalectomy were evaluated based on the Primary Aldosteronism Surgical Outcome (PASO) criteria. In brief, the biochemical outcome was determined by the postoperative aldosterone to renin ratio and serum potassium (K) levels. The clinical outcome was determined by postoperative blood pressure. Biochemical and clinical outcomes were classified as complete, partial, or absent success based on the response to surgery. Details of the outcome criteria have been described previously by Williams et al^18^.

#### Diagnostic modeling

**Figure 1:**
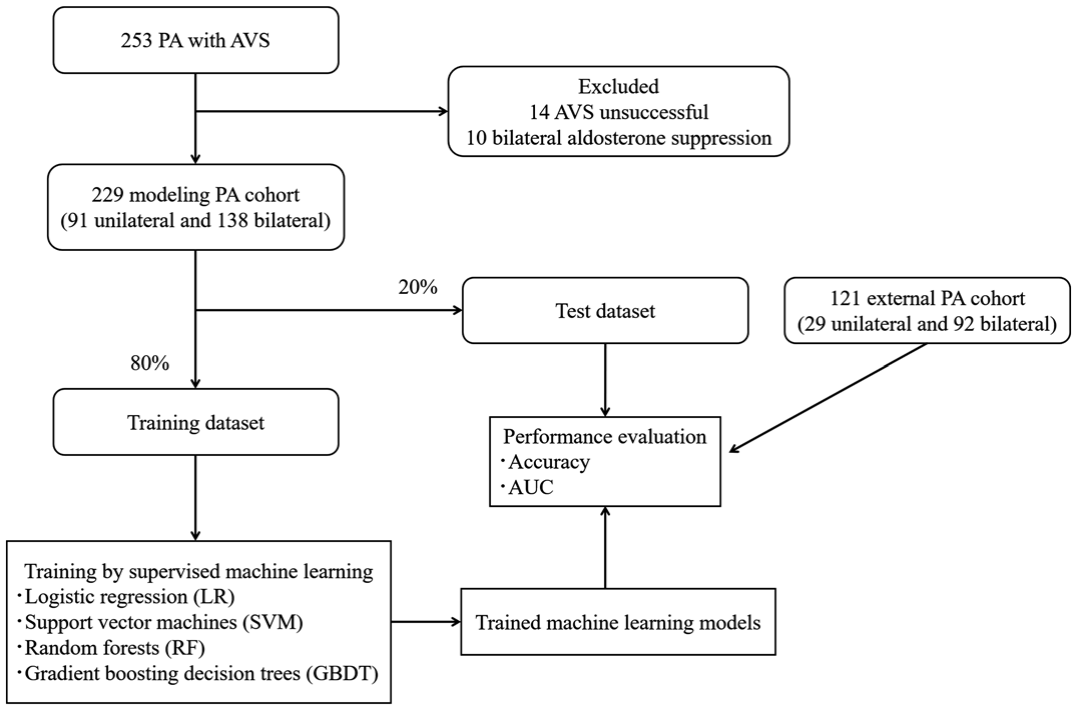
The supervised machine learning workflow of this study.

Python 3.7.6 (library, scikit-learn 0.22.1) was used for the development and validation of diagnostic models made by machine learning techniques^19^. Supervised machine learning classifiers including logistic regression (LR), support vector machines (SVM), random forests (RF), and gradient boosting decision trees (GBDT) were applied to the clinical data. Predictive models for subtype diagnosis of PA were developed using classifiers and were compared between classifiers. We retrospectively investigated the clinical data, including age, body mass index (BMI), systolic blood pressure (SBP), diastolic blood pressure (DBP), and 17 peripheral blood biomarkers; plasma aldosterone concentration (PAC), plasma renin activity (PRA), aspartate aminotransferase (AST), alanine aminotransferase (ALT), albumin (Alb), uric acid (UA), urea nitrogen (UN), estimated glomerular filtration rate (eGFR), total cholesterol (TC), high density lipoprotein cholesterol (HDL-C), low density lipoprotein cholesterol (LDL-C), triglyceride (TG), blood sugar (BS), sodium (Na), the lowest K, chlorine (Cl), and calcium (Ca). In LR and SVM, continuous variables were normalized. To evaluate the accuracy of the models, we stratified randomly split patients with PA to be analyzed into two parts: the training (80%) and test cohorts (20%). Classifiers were trained using stratified tenfold cross-validation of the training cohort. Their resultant predictive performance was evaluated in the test cohort. The hyperparameters of each classifier were adjusted using grid search to optimize the accuracy in the training cohort^20^. In RF, we used the Gini importance as a general measure of feature relevance to confirm the relative ranking of spectral features^21^. The importance of features based on GBDT was also learned from tree models. Each optimized classifier was compared with the subtype predictive accuracy, sensitivity, specificity, and the area under the receiver operating characteristic curve (AUC) for predicting unilateral subtype of PA in the test cohort. The process of supervised machine learning is summarized in Fig. 1. The code that supports the findings of this study is tailored to the data, and is thus not provided since it is of no use as a standalone without access to the data per se.

### Model validation in an external PA cohort

Model generalizability was assessed in an independent sample of patients with PA. An external PA cohort from Sapporo City General Hospital, a single referral center in Japan, was used for validation. We identified 124 patients with PA, who had undergone AVS from March 2017 to December 2019.

### Assay methods

The PAC and PRA were measured using commercial radioimmunoassay kits (SPAC-S Aldosterone Kit; Fuji Rebio, Tokyo, Japan and PRA-FR RIA kit; Fuji Rebio). In the recumbent position, the reference range of PAC was 3.0–15.9 ng/dL and that of PRA was 0.3–2.9 ng/mL/h. All of the other biochemical variables were assayed in plasma or serum by standard methods.

### Statistical analysis

Clinical characteristics were presented as medians with interquartile ranges or as counts with frequencies. Data between two groups were compared using the Mann–Whitney U test and Fisher’s exact test. Pre- and postoperative characteristics were compared using the Wilcoxon signed-rank test. Statistical analysis was performed using Python 3.7.6 (library, SciPy 1.4.1)^22^. A *P* value of less than 0.05 was considered significant.

## Results

### Clinical characteristics of the modeling PA cohort and external PA cohort

We included 229 out of 253 patients with PA in the modeling cohort and excluded 24 patients with unsuccessful AVS (n = 14) and apparent bilateral aldosterone suppression (n = 10) from further analysis. Of 229 patients studied, 91 were diagnosed with unilateral subtype of PA, and the remaining 138 were diagnosed with bilateral subtype according to AVS findings. Clinical and biochemical characteristics are shown in Table 1. Patients with unilateral subtype of PA had a higher PAC and serum Na levels, and a lower PRA, serum Alb, TC, TG, K, and Ca levels than those with bilateral subtype of PA. In addition, the patients were randomly split into two groups with 183 (73 unilateral and 110 bilateral subtypes) and 46 (18 unilateral and 28 bilateral subtypes) patients for the training and test cohorts, respectively. Clinical and biochemical characteristics of the training and test cohorts are shown in Supplementary Table S1. We included 121 out of 124 patients with PA in the external cohort and excluded 3 patients with unsuccessful AVS (n = 1) and apparent bilateral aldosterone suppression (n = 2) from further analysis. Clinical and biochemical characteristics of the modeling and external cohorts are shown in Supplementary Table S2.

**Table 1:** Clinical characteristics of 229 patients with unilateral and bilateral subtypes in the modeling primary aldosteronism cohort.

Variables	Unilateral PA (n = 91)	Bilateral PA (n = 138)	*P* value
Age at diagnosis (years)	53 (43–63)	54 (45–63)	0.504
BMI (kg/m^2^)	24.5 (21.8–27.2)	24.7 (22.3–27.8)	0.537
SBP (mmHg)	140 (130–152)	137 (127–147)	0.133
DBP (mmHg)	88 (80–97)	87 (77–95)	0.595
PAC (ng/dL)	31.3 (21.4–48.3)	16.0 (11.6–21.6)	< 0.001
PRA (ng/mL/h)	0.20 (0.10–0.30)	0.30 (0.20–0.50)	0.001
AST (U/L)	19 (16–24)	20 (16–26)	0.322
ALT (U/L)	17 (12–26)	19 (13–27)	0.473
Alb (g/dL)	4.0 (3.8–4.2)	4.1 (3.9–4.3)	0.018
UA (mg/dL)	5.3 (4.5–6.5)	5.4 (4.6–6.5)	0.776
UN (mg/dL)	13 (10–17)	13 (11–15)	0.689
eGFR (mL/min/1.73m^2^)	76 (64–91)	80 (67–96)	0.189
TC (mg/dL)	190 (161–209)	198 (176–217)	0.011
HDL-C (mg/dL)	49 (39–60)	52 (43–61)	0.091
LDL-C (mg/dL)	111 (91–126)	119 (95–136)	0.070
TG (mg/dL)	103 (70–154)	116 (89–171)	0.036
BS (mg/dL)	95 (88–106)	96 (89–105)	0.822
Na (mEq/L)	143 (141–144)	141 (140–142)	< 0.001
Lowest K (mEq/L)	2.9 (2.6–3.2)	3.7 (3.5–3.9)	< 0.001
Cl (mEq/L)	105 (103–106)	105 (103–106)	0.307
Ca (mg/dL)	9.0 (8.7–9.3)	9.2 (8.9–9.4)	< 0.001

Data are expressed as medians with interquartile ranges or number with percentage. PA indicates primary aldosteronism; BMI, body mass index; SBP, systolic blood pressure; DBP, diastolic blood pressure; PAC, plasma aldosterone concentration; PRA, plasma renin activity; AST, aspartate aminotransferase; ALT, alanine aminotransferase; Alb, albumin; UA, uric acid; UN, urea nitrogen; eGFR, estimated glomerular filtration rate; TC, total cholesterol; HDL-C, high density lipoprotein cholesterol; LDL-C, low density lipoprotein cholesterol; TG, triglyceride; BS, blood sugar; Na, sodium; K, potassium; Cl, chlorine; Ca, calcium.

### Predictive accuracy of subtype diagnosis of PA for the developed models

**Figure 2:**
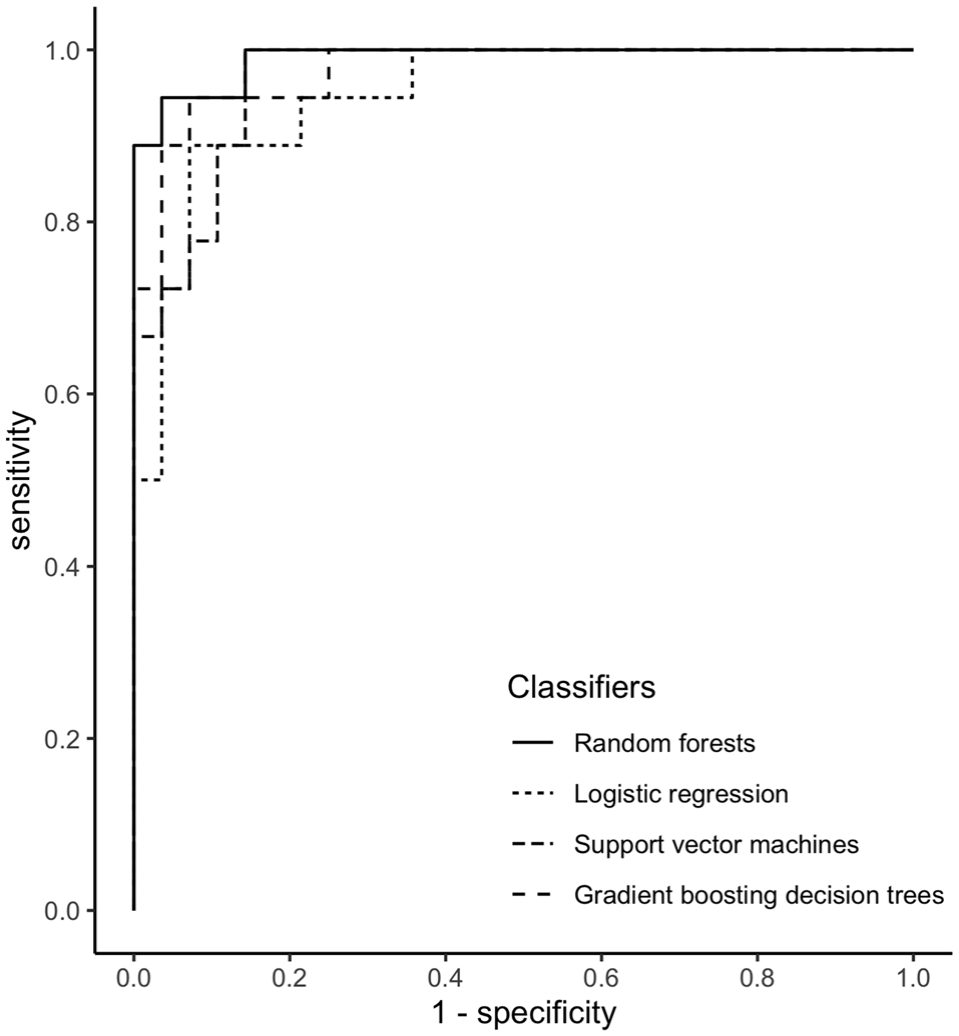
Receiver operating characteristic curves in the test cohort (n = 46) for predicting unilateral subtype of primary aldosteronism in each developed classifier.

**Figure 3:**
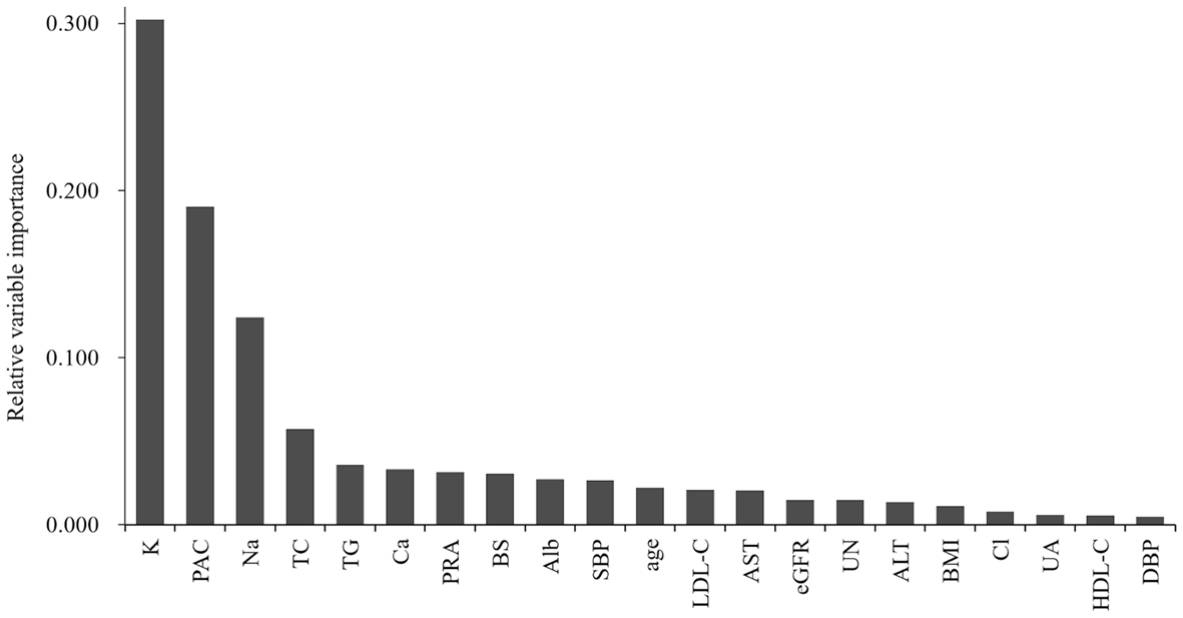
Relative variable importance for predicting unilateral subtype of primary aldosteronism calculated by random forests using 21 clinical data. The relative importance of each variable was arranged in descending order from left to right.

Table 2 and Fig. 2 show the subtype predictive accuracy, sensitivity, specificity, AUC, and receiver operating characteristic curves of unilateral subtype of PA for each classifier using optimum hyperparameters in the test cohort, and Supplementary Table S3 and S4 and Supplementary Figure S1 and S2 show these in the training and external cohorts. Validation in the external cohort was performed in 120 patients with PA, except for one patient whose data on 21 variables were not available. The accuracy provided by developed RF was 95.7% in the test cohort, and was the highest among all the classifiers. A receiver-operation characteristics analysis showed that the AUC for this model were 0.990 (95% confidence interval: 0.971–1.000) in the test cohort. Based on these results, we decided to use RF for subsequent model development. Figure 3 shows the relative Gini importance of each variable of the predictive model in the optimal hyperparameter RF. Serum K, PAC, and serum Na levels were identified as variables of greater relative importance than 0.1 and of high importance in predicting unilateral subtype of PA, in this order. These highly important variables also showed similar trends in GBDT (Supplementary Figure S3). Use of highly correlated variables in LR is known to render the model unstable due to multicollinearity. In fact, there were strong positive correlations between TC and LDL-C, AST and ALT, and moderate positive correlations between SBP and DBP, Alb and Ca, Na and Cl, and moderate negative correlations between eGFR and age or UN, PAC and K, HDL-C and TG among 21 variables. Classifiers other than LR may have had a higher AUC in the test cohort due to the feature of being able to obtain accuracy without suffering from multicollinearity.

**Table 2:** Subtype predictive accuracy and area under the receiver operating characteristic curve of unilateral subtype of primary aldosteronism for each classifier in the test cohort (n = 46).

Classifiers	Accuracy (%)	Sensitivity (%)	Specificity (%)	AUC (95% confidence interval)
LR	89.1	83.3	92.9	0.948 (0.89–1.000)
SVM	84.8	66.7	96.4	0.966 (0.925–1.000)
RF	95.7	94.4	96.4	0.990 (0.971–1.000)
GBDT	87.0	72.2	96.4	0.976 (0.941–1.000)

AUC indicates area under the receiver operating characteristic curve; LR, logistic regression; SVM, support vector machines; RF, random forests; GBDT, and gradient boosting decision trees.

### Predictive accuracy of subtype diagnosis of PA for feature-selected RF

**Figure 4:**
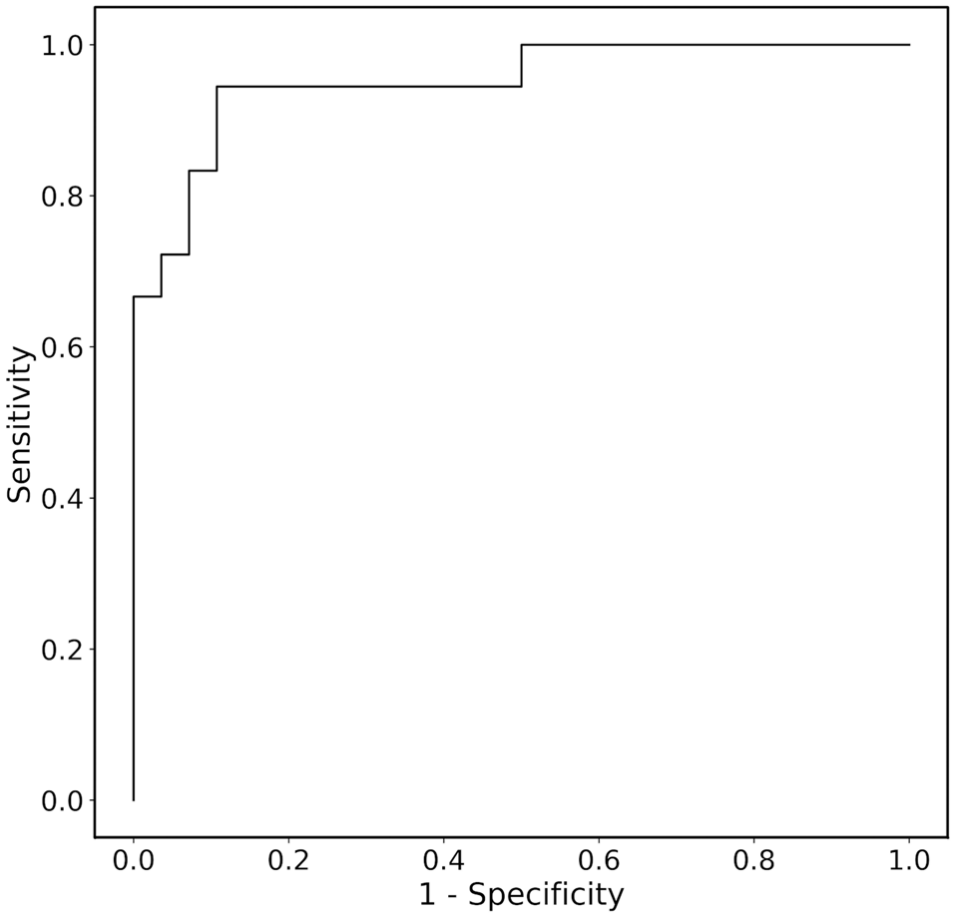
Receiver operating characteristic curve in test dataset (n = 46) for predicting unilateral subtype of primary aldosteronism in feature-selected random forests.

**Figure 5:**
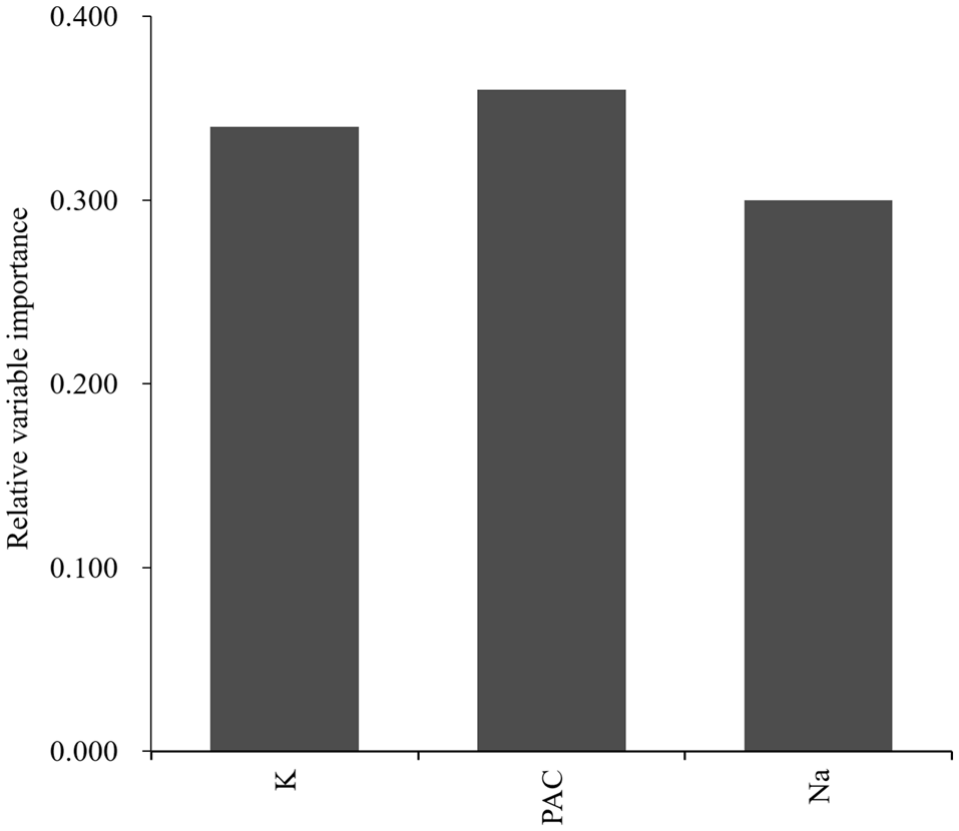
Relative importance of three variables for predicting unilateral subtype of primary aldosteronism in feature-selected random forests.

**Figure 6:**
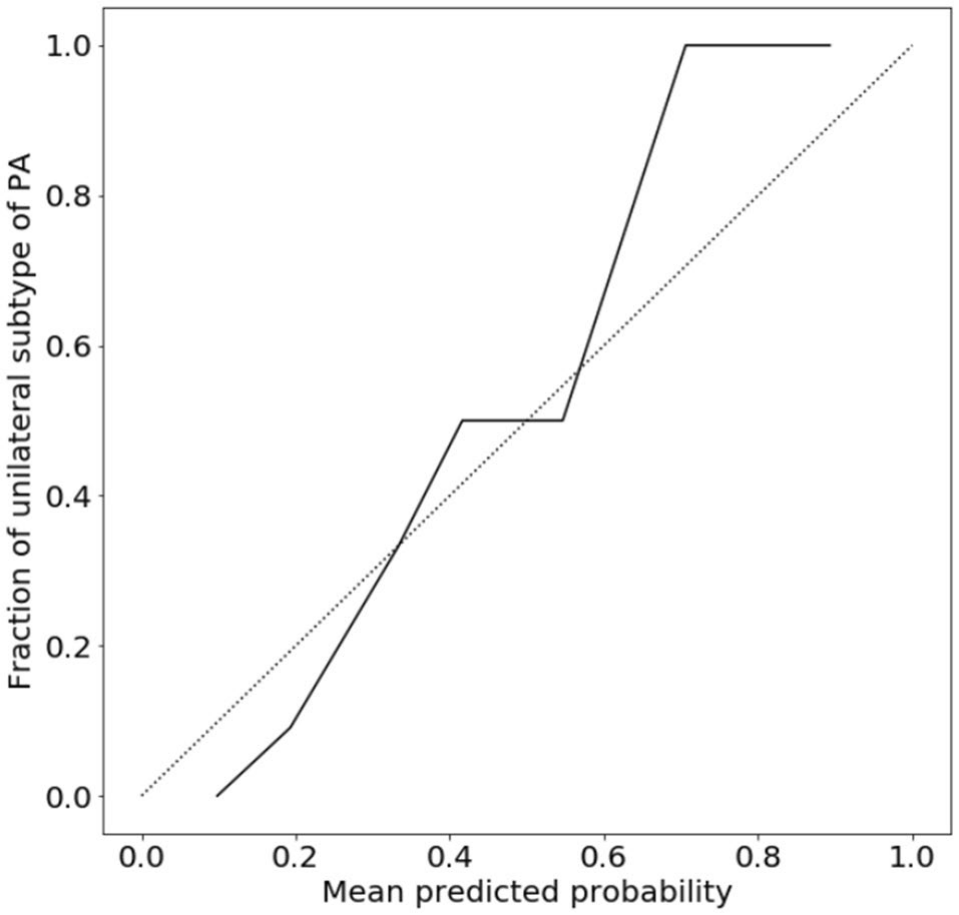
Calibration plot of the feature-selected random forests. The black line represented the plot of the fraction of unilateral subtype of primary aldosteronism versus mean predicted probability. The dotted line represented the calibration line of a perfect model.

Although machine learning is capable of analyzing high-dimensional and multi-variable data, there may not be many variables associated with the predicted probability. Thus, feature selection is important to improve interpretability and shorten learning time^23^. In addition, when adapting predictive models to real-world clinical practice, it is difficult to measure all the predictors in every screening-positive patient. Therefore, we next developed RF model with three important variables; serum K, PAC, and serum Na levels. The hyperparameters were optimized using the same training dataset, and the subtype predictive accuracy and the AUC for the prediction of unilateral subtype of PA were evaluated in the same test dataset. As a result, the accuracy, sensitivity, specificity, and the AUC were 89.1%, 83.3%, 92.9%, and 0.950 (95% confidence interval: 0.888–1.000), respectively. Figures 4 and 5 show the receiver operating characteristic curve and the relative Gini importance of three variables in this RF predictive model, respectively. We ensured that the model predictions were well calibrated (Brier score: 0.11)^24^, as shown in Fig. 6.

### Validation of feature-selected RF in the external PA cohort

The RF model with the three variables was validated using the independent external PA cohort. We included 121 patients with PA in the external cohort for whom variables were available. As a result, the accuracy, sensitivity, specificity, and the AUC were 85.1%, 72.4%, 89.1%, and 0.826 (0.723–0.930), respectively. Furthermore, this accuracy was roughly comparable to that obtained with the test cohort, suggesting that the effect of overfitting in our model was minimal.

### Postoperative changes in three selected variables

We investigated the changes in three selected variables before and after surgical treatment in patients with unilateral subtype of PA to assess whether these variables were associated with the clinical state of unilateral subtype of PA. Of the 91 patients with unilateral subtype of PA in the training and test cohorts, 85 patients underwent adrenalectomy. We analyzed 45 patients, whose data were available between 6 and 12 months, postoperatively. As a result, a significant increase in serum K levels (before: 2.9 [2.6–3.2] mEq/L vs after: 4.4 [4.2–4.6] mEq/L, P < 0.001), and a significant decrease in PAC (before: 32.4 [19.5–54.5] ng/dL vs after: 8.5 [5.2–10.9] ng/dL, P < 0.001) and serum Na levels (before: 143 [141–145] mEq/L vs after: 140 [139–141] mEq/L, P < 0.001) were determined.

### Association between subtype prediction and surgical outcomes

We also examined the surgical outcomes based on PASO criteria of 45 patients whose data were available before and after adrenalectomy. Of 45 patients, 43, 2, and 0 patients achieved complete biochemical success, partial biochemical success, and absent biochemical success, respectively, and 14, 16, and 15 patients achieved complete clinical success, partial clinical success, and absent clinical success, respectively. As shown in Table 3, there was no significant difference in the rate of biochemical and clinical success between 36 patients correctly predicted as unilateral subtype and 9 patients incorrectly predicted as bilateral subtype by feature-selected RF (*P* = 0.364 and 1.000, respectively).

**Table 3:** Surgical outcomes per prediction of feature-selected random forest in 45 patients with unilateral subtype of primary aldosteronism undergoing adrenalectomy.

Model prediction	Biochemical success			Clinical success		
	Complete	Partial	Absent	Complete	Partial	Absent
Unilateral PA (n = 36)	35	1	0	11	13	12
Bilateral PA (n = 9)	8	1	0	3	3	3

PA indicates primary aldosteronism.

## Discussion

In this study, we developed models for predicting the diagnosis of unilateral subtype of PA using routine blood tests based on four supervised machine learning classifiers. Among all the classifiers developed, we found that the RF model had the highest accuracy. Furthermore, we reconstructed a predictive model in the RF with only three contributing blood test parameters, namely PAC, serum K and Na, which are routinely measured and widely available. Notably, hypernatremia and hypokalemia associated with aldosterone hypersecretion were clinical features described when the disease concept of PA was first established by Jerome Conn^25^. We demonstrated that the subtype predictive accuracy was at least 85% in both the test and external PA cohorts. Altogether, these observations suggest that our model has a high predictive value for the surgically curable, unilateral subtype of PA. We suggest that patients who are predicted to have unilateral subtype of PA in general practice should go to appropriate specialized facilities to undergo AVS for lateralization. This is expected to increase the rate of surgical treatment among patients who have undergone AVS. Prediction with three variables, which is easy and inexpensive, may improve the quality of clinical practice of PA in general practice.

Several score-based algorithms and one machine learning model for predicting the subtype diagnosis of PA have been developed. However, all were based on confirmatory test data, computed tomography (CT) findings, or both^26–33^. The previously reported machine learning model was developed with linear discriminant analysis and RF using six variables selected by multivariate analysis, including confirmatory test results and CT findings, and was assumed to be primarily targeted at specialized facilities where AVS is unavailable^33^. Although the algorithms reported previously can be useful in specialized centers, they are not commonly be used for predicting subtype diagnosis of patients with suspected PA in general practice. Relatively young patients with unilateral subtype of PA who did not receive accurate diagnosis and treatment have often been reported to manifest myocardial infarction^34^, aortic dissection^35^, and end-stage renal failure^36^. Early prediction of unilateral PA, which is curable with surgical treatment, is expected to help prevent cardiometabolic complications.

The score-based algorithms reported previously revealed the importance of PAC and serum K levels^26–31,33^. However, for the first time, we demonstrated that serum Na levels were also useful for the subtype diagnosis of PA. It makes sense that serum Na levels are highlighted in our model, taking into account the physiological importance of aldosterone in the regulation of renal Na and K^37^. In addition, patients in this study with unilateral subtype of PA showed a significant decrease in serum Na levels and as well as improvements in PAC and serum K levels after surgical treatment. Therefore, these observations could partially explain the robustness of our machine learning-based model. On the other hand, we found that lipid metabolism and serum Ca levels are of relatively high importance in the predictive models for subtype diagnosis of PA, which is consistent with previous reports that unilateral subtype of PA has significantly lower serum TC and TG levels and a greater degree of secondary hyperparathyroidism relative to bilateral subtype of PA^38,39^. The differences in relative importance of variables other than PAC, serum K, and Na levels were less than 0.05, and the order of these variables would not be strongly associated with subtype diagnosis.

There are a couple of limitations to be addressed. First, this study only retrospectively investigated patients with PA. Further prospective studies are required to assess the validity of our predictive models. Second, subtype diagnosis of PA was based on the AVS results. Although AVS is the standard procedure for subtype classification^8^, the criteria used to determine the lateralization of aldosterone excess are not standardized across centers^40^. Ideally, the subtype diagnosis of PA should be confirmed using postoperative histologic findings and outcomes. Finally, model development and external validation in this study were performed in patients with PA who were referred to Japanese facilities and diagnosed according to the Japanese clinical practice guidelines. The influence of different screening methods and confirmatory tests for PA may have resulted in bias.

In conclusion, we developed and validated predictive models based on machine learning for subtype diagnosis of PA using blood test. The resultant predictive model may increase the opportunities for early diagnosis of unilateral subtype of PA, thereby contributing to the prevention of cardiometabolic complications.

## Supplementary Information

Supplementary Information

## Data Availability

The datasets generated and analyzed during the current study is not publicly available but are available from the corresponding author on reasonable request.
