# Supplementary material for: Machine learning based models for prediction of subtype diagnosis of primary aldosteronism using blood test
Source: Sci Rep. 2021 May 4;11:9140. doi: 10.1038/s41598-021-88712-8 (PMC8096956; doi:10.1038/s41598-021-88712-8)
Supplement: Supplementary file 1 — Supplementary Information [file 41598_2021_88712_MOESM1_ESM.pdf]

Supplementary Information

Machine Learning Based Models for Prediction of Subtype Diagnosis of Primary Aldosteronism Using Blood Test

Hiroki Kaneko[1], Hironobu Umakoshi[1]*, Masatoshi Ogata[1], Norio Wada[2], Norifusa Iwahashi[1], Tazuru Fukumoto[1], Maki Yokomoto-Umakoshi[1], Yui Nakano[1], Yayoi Matsuda[1], Takashi Miyazawa[1], Ryuichi Sakamoto[1], and Yoshihiro Ogawa[1]*

[1]Department of Medicine and Bioregulatory Science, Graduate School of Medical Sciences, Kyushu University, Fukuoka, Japan
[2]Department of Diabetes and Endocrinology, Sapporo City General Hospital, Sapporo, Japan
*Corresponding authors
Hironobu Umakoshi
E-mail: umakoshi@med.kyushu-u.ac.jp
Yoshihiro Ogawa
E-mail: yogawa@med.kyushu-u.ac.jp

Supplementary Table S1. Clinical characteristics of 229 patients in the modeling primary aldosteronism cohort

Variables	Training cohort (n = 183)	Test cohort (n = 46)	P value
Unilateral PA, n (%)	73 (39.9)	18 (39.1)	1.000
Age at diagnosis (years)	54 (45-63)	53 (42-63)	0.761
BMI (kg/m^2)	24.8 (22.4-27.8)	23.9 (21.6-26.9)	0.391
SBP (mmHg)	139 (130-148)	139 (125-150)	0.605
DBP (mmHg)	88 (79-96)	86 (78-94)	0.687
PAC (ng/dL)	19.6 (13.3-32.2)	20.9 (15.5-27.2)	0.927
PRA (ng/mL/h)	0.30 (0.20-0.40)	0.30 (0.20-0.48)	0.487
AST (U/L)	20 (16-25)	20 (15-24)	0.790
ALT (U/L)	19 (13-26)	17 (12-30)	0.415
Alb (g/dL)	4.0 (3.9-4.3)	4.1 (3.8-4.3)	0.526
UA (mg/dL)	5.4 (4.5-6.5)	5.2 (4.6-6.1)	0.332
UN (mg/dL)	13 (11-16)	12 (10-16)	0.630
eGFR (mL/min/1.73m^2)	78 (65-93)	80 (71-100)	0.116

TC (mg/dL)	194 (173-214)	194 (170-218)	0.949
HDL-C (mg/dL)	49 (42-61)	52 (43-65)	0.584
LDL-C (mg/dL)	114 (94-130)	111 (95-135)	0.726
TG (mg/dL)	113 (80-171)	110 (86-157)	0.903
BS (mg/dL)	96 (88-105)	97 (90-110)	0.486
Na (mEq/L)	142 (140-143)	141 (140-143)	0.197
Lowest K (mEq/L)	3.5 (3.0-3.8)	3.6 (2.8-3.7)	0.544
Cl (mEq/L)	105 (103-106)	104 (102-105)	0.053
Ca (mg/dL)	9.1 (8.9-9.3)	9.1 (8.9-9.4)	0.583

Data are expressed as medians with interquartile ranges or number with percentage. PA, primary aldosteronism; BMI, body mass index; SBP, systolic blood pressure; DBP, diastolic blood pressure; PAC, plasma aldosterone concentration; PRA, plasma renin activity; AST, aspartate aminotransferase; ALT, alanine aminotransferase; Alb, albumin; UA, uric acid; UN, urea nitrogen; eGFR, estimated glomerular filtration rate; TC, total cholesterol; HDL-C, high density lipoprotein cholesterol; LDL-C, low density lipoprotein cholesterol; TG, triglyceride; BS, blood sugar; Na, sodium; K, potassium; Cl, chlorine; Ca, calcium.

Supplementary Table S2. Patient clinical characteristics of the modeling and external primary aldosteronism cohort

Variables	Modeling PA cohort (n = 229)	External PA cohort (n = 121)	P value
Unilateral PA, n (%)	91 (39.7)	29 (24.0)	0.003
Age at diagnosis (years)	54 (45-63)	50 (44-56)	0.005
BMI (kg/m^2)	24.6 (22.1-27.7)	24.8 (22.5-26.6)	0.669
SBP (mmHg)	139 (129-148)	137 (130-150)	0.963
DBP (mmHg)	88 (78-96)	89 (80-96)	0.370
PAC (ng/dL)	19.7 (13.6-30.1)	18.1 (13.2-26.3)	0.208
PRA (ng/mL/h)	0.30 (0.20-0.40)	0.20 (0.20-0.50)	0.129
AST (U/L)	20 (16-25)	19 (17-24)	0.964
ALT (U/L)	18 (13-27)	18 (13-25)	0.916
Alb (g/dL)	4.0 (3.8-4.3)	4.2 (4.1-4.4)	<0.001
UA (mg/dL)	5.3 (4.5-6.5)	5.5 (4.4-6.3)	0.620
UN (mg/dL)	13 (11-16)	13 (10-15)	0.105
eGFR (mL/min/1.73m^2)	78 (66-94)	87 (73-99)	0.003

TC (mg/dL)[a]	194 (171-215)	198 (180-220)	0.139
HDL-C (mg/dL)[a]	50 (42-61)	59 (46-68)	<0.001
LDL-C (mg/dL)[a]	114 (94-133)	113(97-131)	0.998
TG (mg/dL)[a]	112 (80-167)	103 (70-138)	0.048
BS (mg/dL)	96 (89-105)	100 (94-108)	0.003
Na (mEq/L)	142 (140-143)	142 (141-143)	<0.001
Lowest K (mEq/L)	3.5 (3.0-3.8)	3.8 (3.5-4.0)	<0.001
Cl (mEq/L)	105 (103-106)	106 (104-107)	<0.001
Ca (mg/dL)	9.1 (8.9-9.3)	9.4 (9.2-9.6)	<0.001

Data are expressed as medians with interquartile ranges or number with percentage. PA, primary aldosteronism; BMI, body mass index; SBP, systolic blood pressure; DBP, diastolic blood pressure; PAC, plasma aldosterone concentration; PRA, plasma renin activity; AST, aspartate aminotransferase; ALT, alanine aminotransferase; Alb, albumin; UA, uric acid; UN, urea nitrogen; eGFR, estimated glomerular filtration rate; TC, total cholesterol; HDL-C, high density lipoprotein cholesterol; LDL-C, low density lipoprotein cholesterol; TG, triglyceride; BS, blood sugar; Na, sodium; K, potassium; Cl, chlorine; Ca, calcium.

[a]One patient (external PA cohort) with no data available was excluded from this analysis.

Table S3. Subtype predictive accuracy and area under the receiver operating characteristic curve of unilateral subtype of primary aldosteronism for each classifier in the training cohort (n = 183)

Classifiers	Accuracy (%)	Sensitivity (%)	Specificity (%)	AUC (95% confidence interval)
LR	88.5	86.3	90.0	0.925 (0.886-0.965)
SVM	85.8	75.3	92.7	0.939 (0.902-0.975)
RF	89.6	84.9	92.7	0.959 (0.933-0.986)
GBDT	92.3	83.6	98.2	0.965 (0.939-0.991)

AUC indicates area under the receiver operating characteristic curve; LR, logistic regression; SVM, support vector machines; RF, random forests; GBDT, gradient boosting decision trees.

Table S4. Subtype predictive accuracy and area under the receiver operating characteristic curve of unilateral subtype of primary aldosteronism for each classifier in the external cohort (n = 120)

Classifiers	Accuracy (%)	Sensitivity (%)	Specificity (%)	AUC (95% confidence interval)
LR	88.3	69.0	94.5	0.877 (0.794-0.961)
SVM	88.3	65.5	95.6	0.875 (0.786-0.964)
RF	88.3	69.0	94.5	0.872 (0.791-0.952)
GBDT	88.3	69.0	94.5	0.848 (0.752-0.944)

AUC indicates area under the receiver operating characteristic curve; LR, logistic regression; SVM, support vector machines; RF, random forests; GBDT, gradient boosting decision trees.

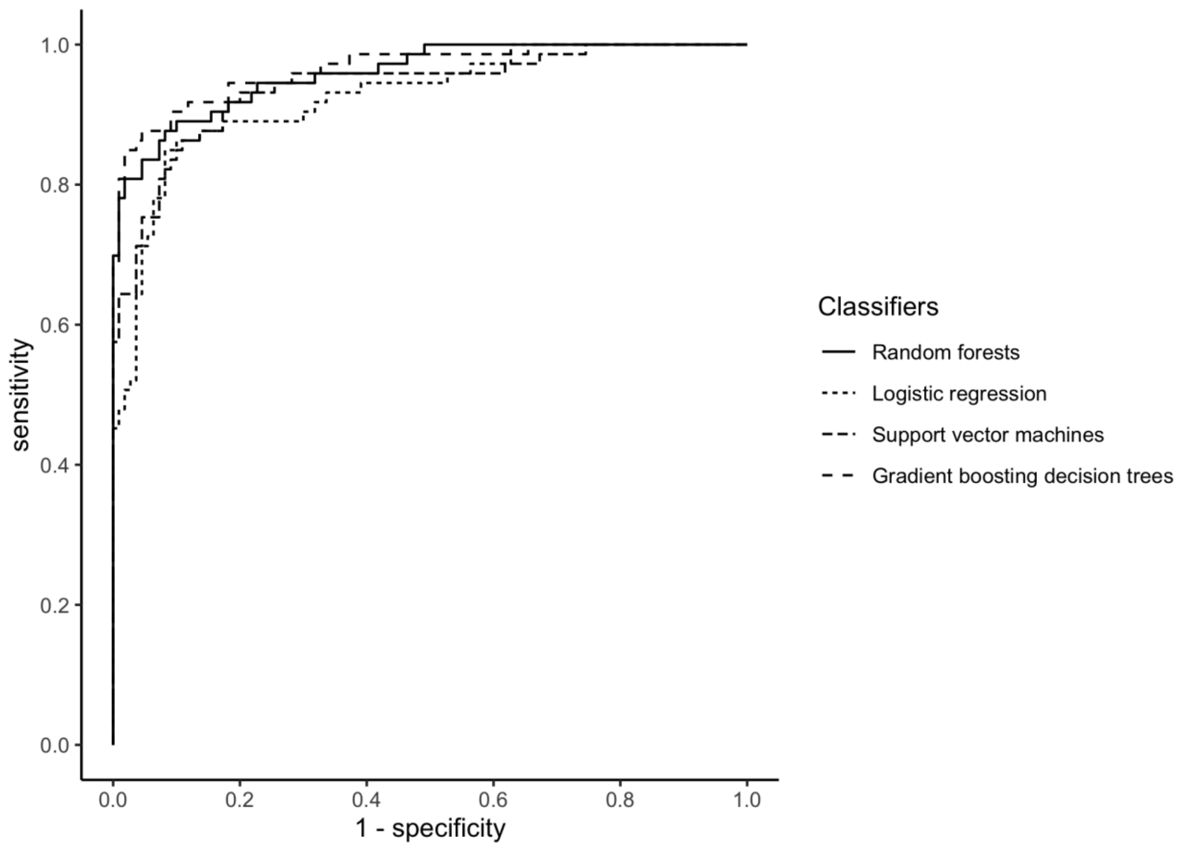

Supplementary Figure S1. Receiver operating characteristic curves in the training cohort (n = 183) for predicting unilateral subtype of primary aldosteronism in each developed classifier.

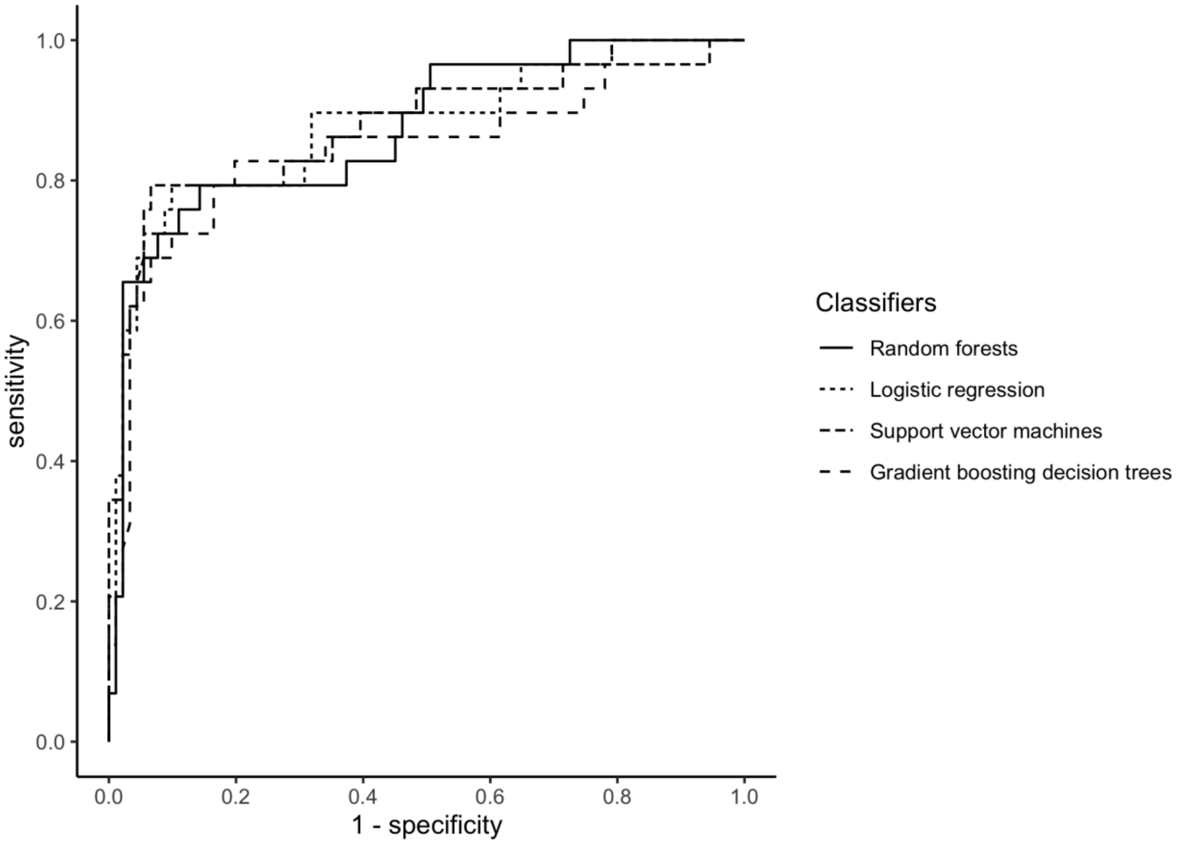

Supplementary Figure S2. Receiver operating characteristic curves in the external cohort (n = 120) for predicting unilateral subtype of primary aldosteronism in each developed classifier.

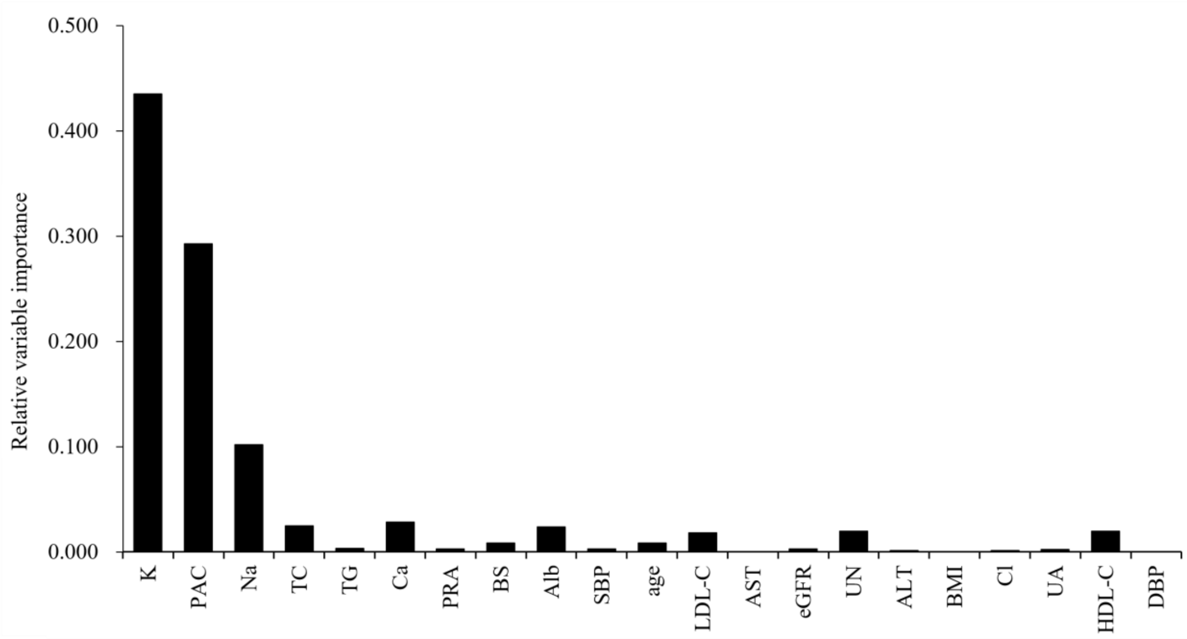

Supplementary Figure S3. Relative importance of 21 variables for predicting unilateral subtype of primary aldosteronism calculated by gradient boosting decision trees. The relative importance of each variable was arranged in the same order as in Figure 3.
